# Supplementary material for: Cooperation between a root fungal endophyte and host‐derived coumarin scopoletin mediates Arabidopsis iron nutrition
Source: New Phytol. 2025 Aug 20;248(2):857–71. doi: 10.1111/nph.70476 (PMC12445806; doi:10.1111/nph.70476)
Supplement: Supplementary file 2 — Fig. S1 Heat‐killed fungal endophyte F80 does not improve Arabidopsis growth under iron‐limited conditions. Fig. S2 The concentration of mineral elements in shoots measured by ICP‐MS. Fig. S3 Phenotypes of mutants disrupted in coumarin biosynthesis or reductive iron uptake under available iron conditions in the presence or not of the fungal endophyte F80. Fig. S4 Changes in metabolite profile of M. phaseolina F80 cultures when supplemented with scopoletin. Fig. S5 Coumarin supplementation does not rescue fro2, while Na2EDTA supplementation rescues f6'h1 but not fro2. Table S1 Medium composition of ARE and vitamin solutions used in 96‐well fungal culture assay. Please note: Wiley is not responsible for the content or functionality of any Supporting Information supplied by the authors. Any queries (other than missing material) should be directed to the New Phytologist Central Office. [file NPH-248-857-s002.docx]

## *New Phytologist* Supporting Information

Article title: **Cooperation between a root fungal endophyte and host-derived coumarin scopoletin mediates *Arabidopsis* iron nutrition**

Authors: Lara Van Dijck^1^, Dario Esposto^2^, Charlotte Huelsmann^1^, Milena Malisic^1,3^, Anthony Piro^1,3^, Ricardo F. H. Giehl^4^, Gerd U. Balcke^2^, Alain Tissier^2^, Jane E. Parker^1,3^ *

Article acceptance date: 28 July 2025

The following Supporting Information is available for this article:

**Fig. S1** Heat-killed fungal endophyte F80 does not improve *Arabidopsis* growth under iron-limited conditions.

**Fig. S2** The concentration of mineral elements in shoots measured by ICP-MS.

**Fig. S3** Phenotypes of mutants disrupted in coumarin biosynthesis or reductive iron uptake under available iron conditions in the presence or not of the fungal endophyte F80.

**Fig. S4** Changes in metabolite profile of *M. phaseolina* F80 cultures when supplemented with scopoletin.

**Fig. S5** Coumarin supplementation does not rescue *fro2* while Na_2_EDTA supplementation rescues *f6’h1*, but not *fro2*.

**Table S1** Medium composition of ARE and vitamin solutions used in 96-Well fungal culture assay.

**Dataset S1** Raw data excel file [provided as a separate Excel file]


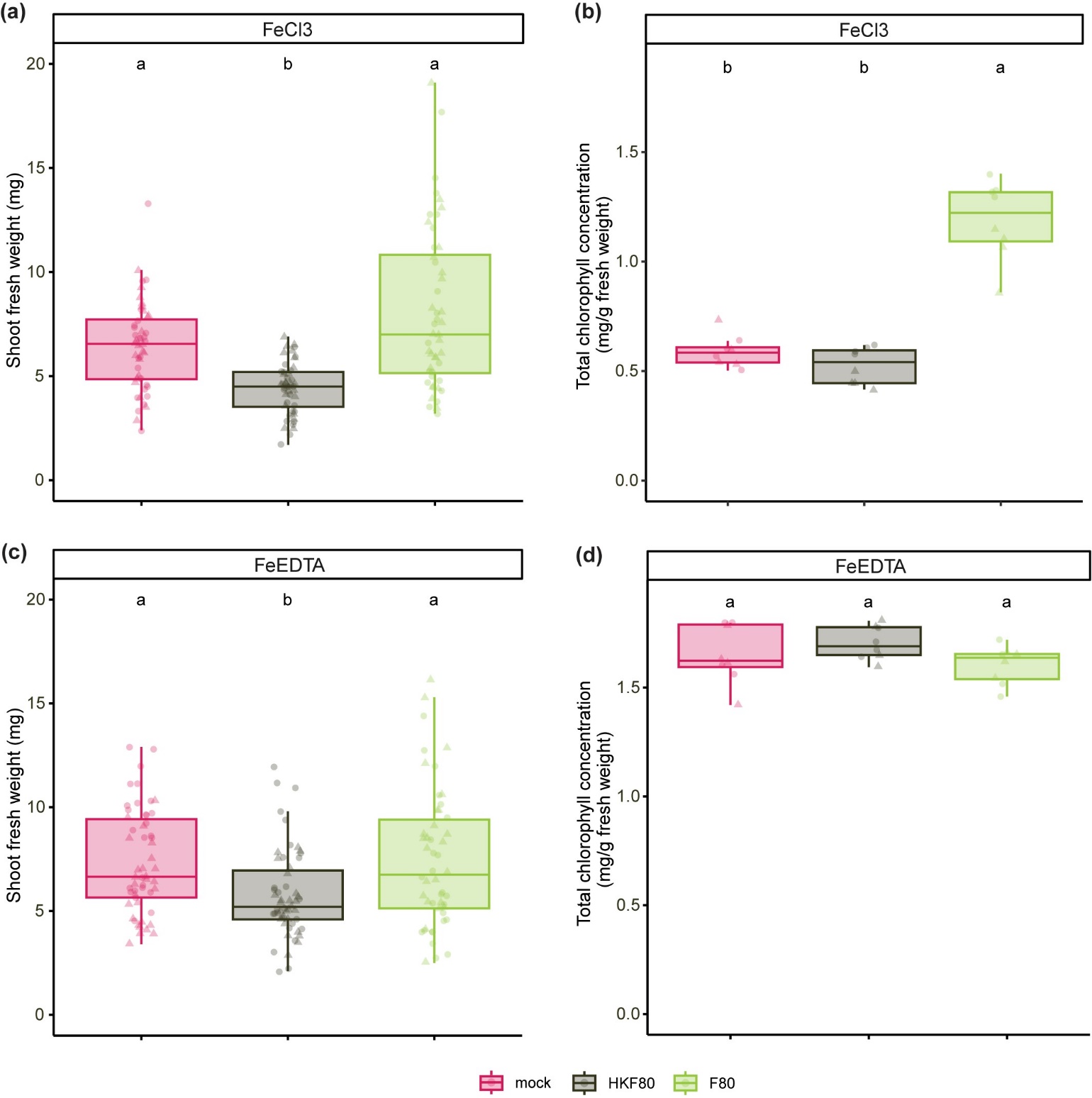


**Fig. S1 Heat-killed fungal endophyte F80 does not improve *Arabidopsis* growth under iron-limited conditions.**

(a,c) SFW and (b,d) shoot chlorophyll concentration at 2 weeks of growth after transfer. 7-day-old Col-0 seedlings were transferred to half-strength MS medium with unavailable iron (50 µM FeCl_3_) (a,b) or available iron (50 µM FeEDTA) (c,d) at pH 5.7 mock or inoculated with heat-killed (HK) or live F80. Letters indicate signiﬁcant pairwise differences between groups (*p*-adj≤0.05) by a Dunn pairwise comparison test with Benjamini-Hochberg correction. Data are from two full factorial replicates (represented by different shapes).


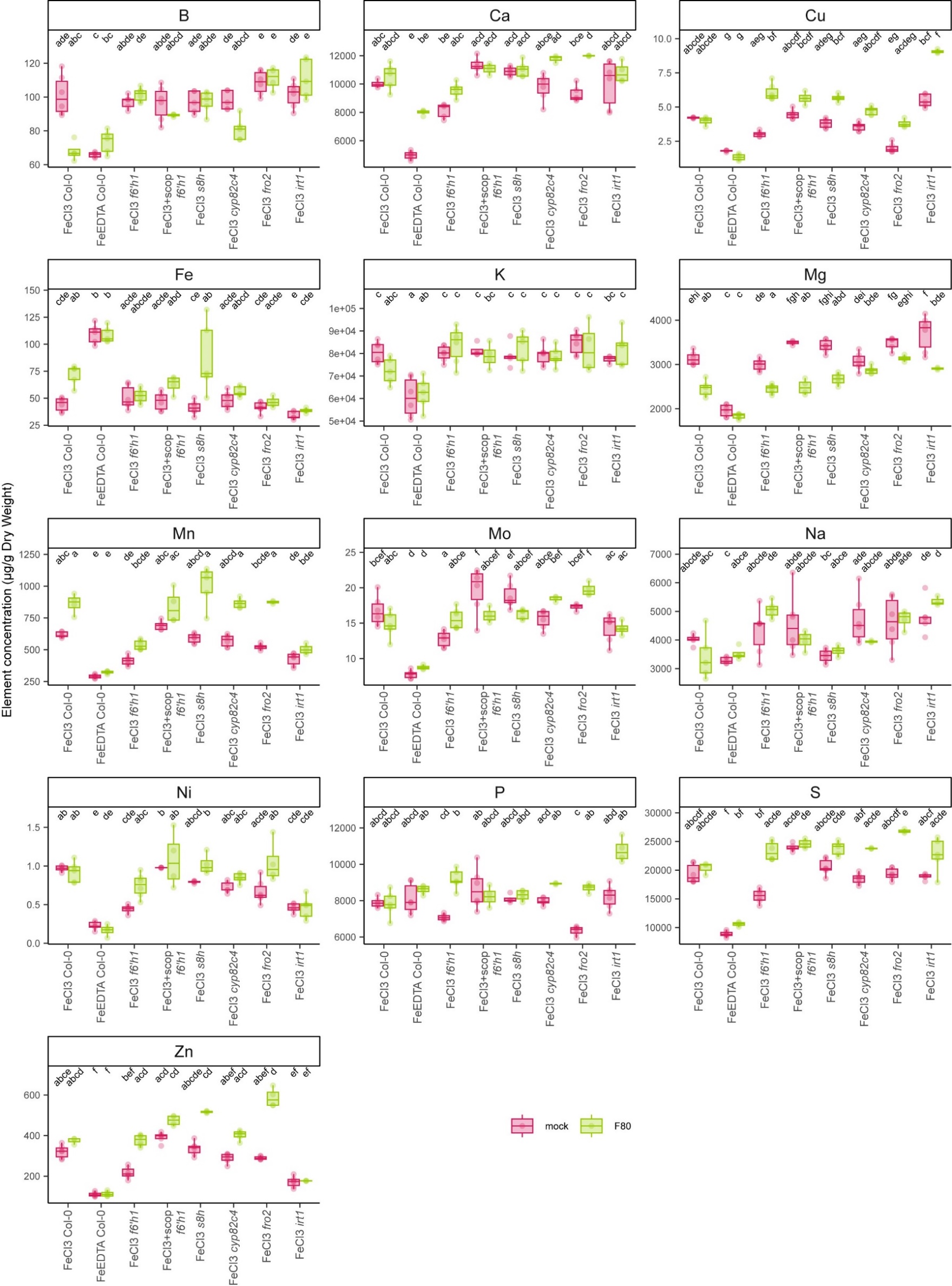


**Fig. S2 Concentrations of mineral elements in shoots measured by ICP-MS.**

Shoot mineral element concentrations at 2 weeks of growth after transfer. 7-day-old seedlings were transferred to half-strength MS medium at pH 5.7. Each plot depicts (from left to right) Col-0 grown on 50 µM FeCl_3_, Col-0 grown on 50 µM FeEDTA, *f6’h1* grown on 50 µM FeCl_3_, *f6’h1* grown on 50 µM FeCl_3_ supplemented with 10 µM scopoletin, *s8h* grown on 50 µM FeCl_3_, *cyp82c4* grown on 50 µM FeCl_3_, *fro2* grown on 50 µM FeCl_3_ and *irt1* grown on 50 µM FeCl_3_. Letters indicate signiﬁcant pairwise differences between groups (*p*-adj≤0.05) by a Tukey’s HSD corrected for multiple comparisons when the data were normally distributed or by a Dunn pairwise comparison test with Benjamini-Hochberg correction when not normally distributed. Data are from three full factorial replicates.

**
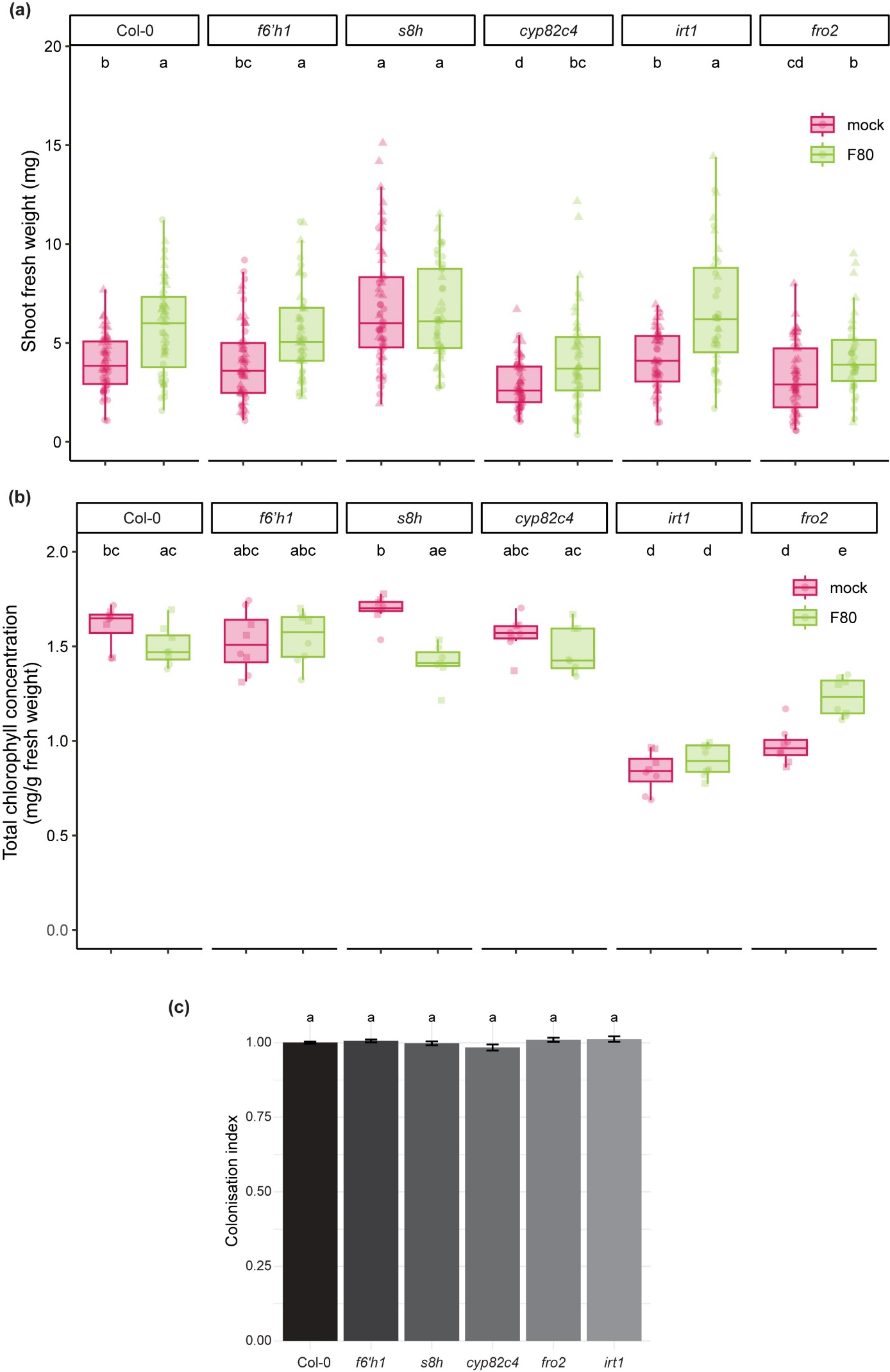
**

**Fig. S3 Phenotypes of mutants disrupted in coumarin biosynthesis or reductive iron uptake under available iron conditions in the presence or not of the fungal endophyte F80.**

(a) SFW and (b) shoot chlorophyll content at 2 weeks of growth after transfer of indicated mutants in the coumarin biosynthesis pathway and the iron reductive import mechanism. 7-day-old seedlings were transferred to half-strength MS medium with available iron (50 µM FeEDTA) at pH 5.7 mock or inoculated with F80. Letters indicate signiﬁcant pairwise differences between groups (*p*-adj≤0.05) by a Dunn pairwise comparison test with Benjamini-Hochberg correction. Data are from two full factorial replicates (represented by different shapes). (c) F80 colonisation index normalised to Col-0 control, corresponding to experiment shown in Figure 2b,c. Letters indicate signiﬁcant pairwise differences between groups (*p*-adj≤0.05) by a Tukey’s HSD corrected for multiple comparisons.


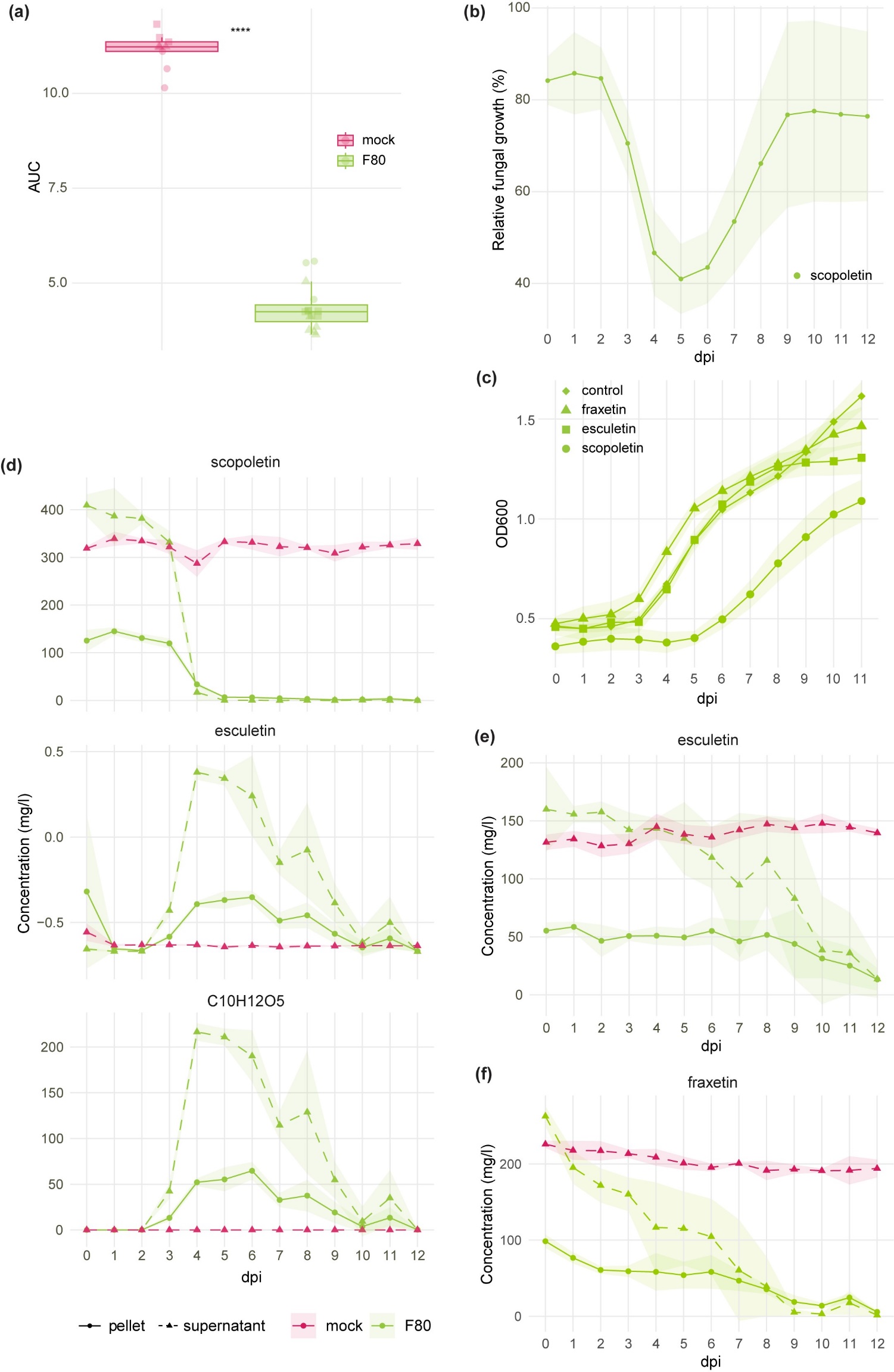


**Fig. S4 Metabolite profiles of *M. phaseolina* F80 cultures when supplemented with scopoletin.**

(a) Area Under the Curve (AUC) of the scopoletin detection plot in Figure 3A. Specific scopoletin fluorescence measured in *in vitro* mock and F80 cultures supplemented with 2 mM scopoletin (excitation at 385nm and emission detected at 470nm). (b) The mean OD600 of F80 cultures in 2 mM scopoletin relative to the OD600 of cultures in the absence of scopoletin from 0-12 dpi. Data combined from 3 independent biological experiments. The curve-shade indicates the standard error. (c) Mean OD600 of F80 cultures in the absence of coumarins (control, diamond), 2 mM esculetin (square), fraxetin (triangle) or scopoletin (circle) from 0-11 dpi. Data are combined from two independent biological experiments. Shading around the curves indicates standard error. (d) Absolute quantification of the MS-QTOF-IDA-MS/MS peak area data shown in Figure 3c of the indicated compounds in F80 culture supernatant (dashed line) and the fungal pellet (full line) grown for 0-12 dpi supplemented with 2 mM scopoletin. Curve-shade indicates the standard deviation. (e) Absolute quantification of the MS-QTOF-IDA-MS/MS peak area data shown in Figure 3c of esculetin in F80 culture supernatant (dashed line) and the fungal pellet (full line) grown for 0-12 dpi supplemented with 2 mM esculetin. Curve-shade indicates the standard deviation. (f) Absolute quantification of the MS-QTOF-IDA-MS/MS peak area data shown in Figure 3d of fraxetin in F80 culture supernatant (dashed line) and the fungal pellet (full line) grown for 0-12 dpi supplemented with 2mM fraxetin. Curve-shade indicates the standard deviation.


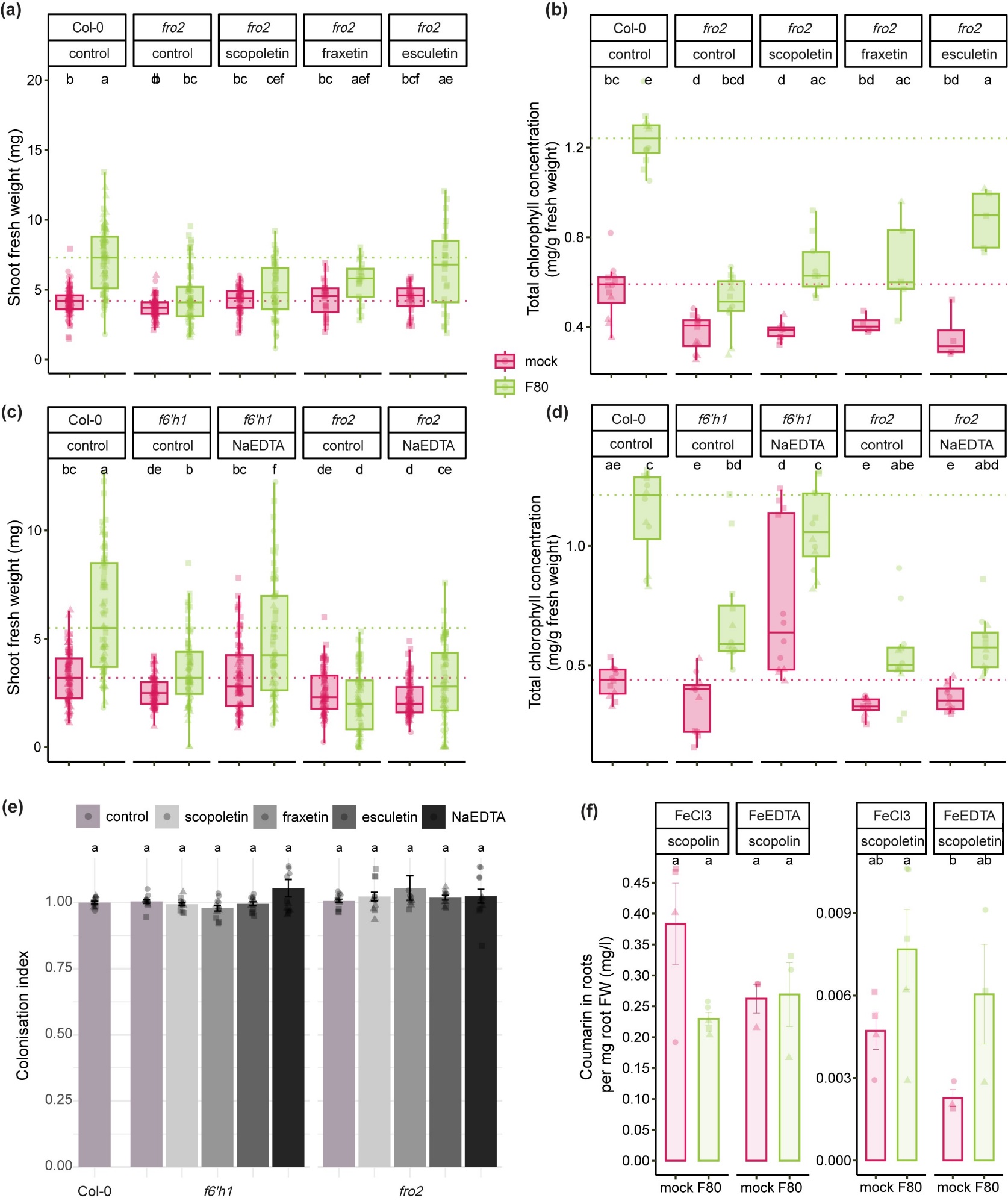


**Fig. S5 Coumarin supplementation does not rescue *fro2* while Na_2_EDTA supplementation rescues *f6’h1*, but not *fro2*.**

(a,c) SFW and (b,d) shoot chlorophyll concentration at 2 weeks of growth after transfer. 7-day-old Col-0, *f6’h1* and *fro2* seedlings were transferred to half-strength MS medium with unavailable iron (50 µM FeCl3) at pH 5.7, supplemented with 10 µM of scopoletin, fraxetin, esculetin or Na_2_EDTA (indicated above the graphs) or an equal amount of DMSO (control), mock or inoculated with F80. Dashed line indicates the mean of Col-0. Letters indicate signiﬁcant pairwise differences between groups (*p*-adj≤0.05) by a Dunn pairwise comparison test with Benjamini-Hochberg correction. Data are from three full factorial replicates (represented by different shapes). (e) F80 colonisation index normalised to Col-0, corresponding to experiment shown in Figure 4 (a,b) and S4 (a,b,c,d). Letters indicate signiﬁcant pairwise differences between groups (*p*-adj≤0.05) by a Tukey’s HSD corrected for multiple comparisons. (f) The concentration of scopoline and scopoletin in roots 1 week past transfer of 9-day-old seedlings to half-strength MS medium with unavailable iron (50 µM FeCl3) or available (50 µM FeEDTA) at pH 5.7 mock or inoculated with F80. Approximately 150-250 mg of roots pooled from 10 plates were used to extract metabolites and analysed with MS-QTOF-IDA-MS/MS. Bars represent the mean from 3 biological replicates (n=1-2) with standard error bars. Letters indicate signiﬁcant pairwise differences between groups (*p*-adj≤0.05) by a Tukey’s HSD corrected for multiple comparisons.

| **5X ARE (artificial root exudates)** | |
| --- | --- |
| **compound** | **g/l** |
| Glucose | 8.2 |
| Fructose | 8.2 |
| Saccharose | 4.2 |
| Citric Acid | 3.2 |
| Lactic Acid | 3.2 |
| Succinic Acid | 4.6 |
| Alanine | 4 |
| Serine | 4.8 |
| Glutamic Acid | 4 |
| **100X Vitamins** | |
| **compound** | **mg/l** |
| thiamine-HCl | 10.12 |
| nicotinic acid | 12.31 |
| folic acid | 1.99 |
| pyridoxine hydrochloride | 20.56 |
| 4-aminobenzoic acid | 4.11 |
| Calcium D pantothenate | 4.77 |
| biotin | 1.00 |

**Table S1 medium composition of ARE and vitamin solutions used in the 96-Well fungal culture assay.**
